# Depression as a mediator between hearing impairment and cognitive function among older couples

**DOI:** 10.1093/geroni/igaf122.2931

**Published:** 2025-12-31

**Authors:** Suyeong Bae, Chang Dae Lee, Ji-Hyuk Park

**Affiliations:** Yonsei University Mirae Campus, Wonju, Republic of Korea; Indiana University Indianapolis, School of Health & Human Sciences, Indianapolis, Indiana, United States; Yonsei University Mirae Campus, Wonju, Republic of Korea

## Abstract

Our study investigated how depression mediated the relationship between hearing impairment and cognitive function in older couples, addressing previous limitations by focusing on partner interdependence. This secondary data analysis used the 2023 Korea Elderly Survey. Hearing impairment, measured by a self-reported item regarding hearing inconvenience, was the independent variable. The dependent variable was cognitive function measured by the Korean-Mini Mental State Examination-Second edition, and the mediator was depression, assessed by the Korean version of the short Geriatric Depression Scale. We utilized the actor-partner interdependence mediation model (APIMeM) to examine the association between hearing impairment and cognitive function, as well as the mediating effect of depression in the context of interdependence. Our sample consisted of 2,233 older adult couples. The mean age was 71.65±5.57 years for females and 74.62±6.11 years for males. Based on the APIMeM model, an individual’s hearing impairment was significantly associated with their own cognitive function (males: coefficient = 1.93; females: coefficient = 2.02) and their partners’ cognitive function (males: coefficient = 0.84; females: coefficient = 1.37). Additionally, an individual’s cognitive function was significantly associated with the partner’s hearing impairment, and this association was mediated by the individual’s own depressive symptoms for both males (coefficient = 0.22) and females (coefficient = 0.25). Our findings highlight the mediating role of depression in the association between hearing impairment and cognitive function in older couples, considering both individual and interdependent effects. These results provided evidence for considering partner interdependence when addressing cognitive health in later life.

